# Antithrombotic Agents and Cancer

**DOI:** 10.3390/cancers10080253

**Published:** 2018-07-31

**Authors:** Annalisa Bruno, Melania Dovizio, Stefania Tacconelli, Annalisa Contursi, Patrizia Ballerini, Paola Patrignani

**Affiliations:** Department of Neuroscience, Imaging and Clinical Sciences and Center for Research on Aging and Translational Medicine (CeSI-MeT), “G. d’Annunzio” University of Chieti, 66100 Chieti, Italy; a.bruno@unich.it (A.B.); m.dovizio@unich.it (M.D.); s.tacconelli@unich.it (S.T.); annalisa.contursi@unich.it (A.C.); patrizia.ballerini@unich.it (P.B.)

**Keywords:** antiplatelet drugs, cancer, platelets, metastasis, epithelial-mesenchymal transition, immune surveillance

## Abstract

Platelet activation is the first response to tissue damage and, if unrestrained, may promote chronic inflammation-related cancer, mainly through the release of soluble factors and vesicles that are rich in genetic materials and proteins. Platelets also sustain cancer cell invasion and metastasis formation by fostering the development of the epithelial-mesenchymal transition phenotype, cancer cell survival in the bloodstream and arrest/extravasation at the endothelium. Furthermore, platelets contribute to tumor escape from immune elimination. These findings provide the rationale for the use of antithrombotic agents in the prevention of cancer development and the reduction of metastatic spread and mortality. Among them, low-dose aspirin has been extensively evaluated in both preclinical and clinical studies. The lines of evidence have been considered appropriate to recommend the use of low-dose aspirin for primary prevention of cardiovascular disease and colorectal cancer by the USA. Preventive Services Task Force. However, two questions are still open: (i) the efficacy of aspirin as an anticancer agent shared by other antiplatelet agents, such as clopidogrel; (ii) the beneficial effect of aspirin improved at higher doses or by the co-administration of clopidogrel. This review discusses the latest updates regarding the mechanisms by which platelets promote cancer and the efficacy of antiplatelet agents.

## 1. Introduction

Antithrombotic agents mainly comprise two classes of drugs that are commonly used in clinical practice to combat pathologic thrombosis: antiplatelet drugs and anticoagulants. They share the common action of preventing a clot from forming and growing, but it is accomplished via different mechanisms [1]. The antiplatelet drugs affect platelet aggregation (clumping), while the anticoagulants manipulate the blood coagulation process through the reduction of fibrin formation [1]. Antithrombotic agents are the first choice for the prevention and treatment of cardiovascular (CV) disease [2]. Moreover, the use of anticoagulants and antiplatelet drugs is included in the armamentarium to combat cancer and/or its clinical consequences [3]. Anticoagulants are used for the prevention of venous thromboembolic events that are associated with cancer [4]. Preclinical studies have reported that anticoagulants (unfractionated heparin (UFH), low molecular weight heparins (LMWH), and the Xa inhibitor fondaparinux) may affect the platelet release of angiogenic proteins through the inhibition of thrombin-dependent protease-activated receptor1 (PAR1) activation [5]. This mechanism might play a role in the anticancer effects of UFH and LMWH, independent of their anticoagulant functions, found in preclinical studies [6]. These findings, together with the efficacy of antiplatelet agents, such as aspirin (acetylsalicylic acid) at low doses, to reduce death and incidence of cancer [7,8,9,10,11], confirm the central role of platelets in tumorigenesis and metastasis development. This review discusses the latest updates regarding the mechanisms by which platelets promote cancer and the efficacy of antiplatelet agents.

## 2. The Roles of Activated Platelets in Cancer

Platelets are anucleate cell fragments that play a pivotal role in hemostasis and thrombosis [12]. However, numerous pieces of evidence show novel functions of platelets, including immune response, inflammation and metastasis formation, which are mediated by the capacity of platelets to interact and activate other cells types via a direct contact and/or the release of different soluble mediators, including lipids (such as, prostaglandin (PG)E_2_ and thromboxane (TX)A_2_) and proteins (such as, stromal cell-derived factor (SDF)-1α, growth and angiogenic factors, cytokines and chemokines), and extracellular vesicles (EVs), such as exosomes and microparticles (MPs) [13,14,15]. EVs promote the intercellular communication by facilitating the exchange of biological materials (including mRNAs and microRNAs (miRs)) between cells [13].

**Figure 1:**
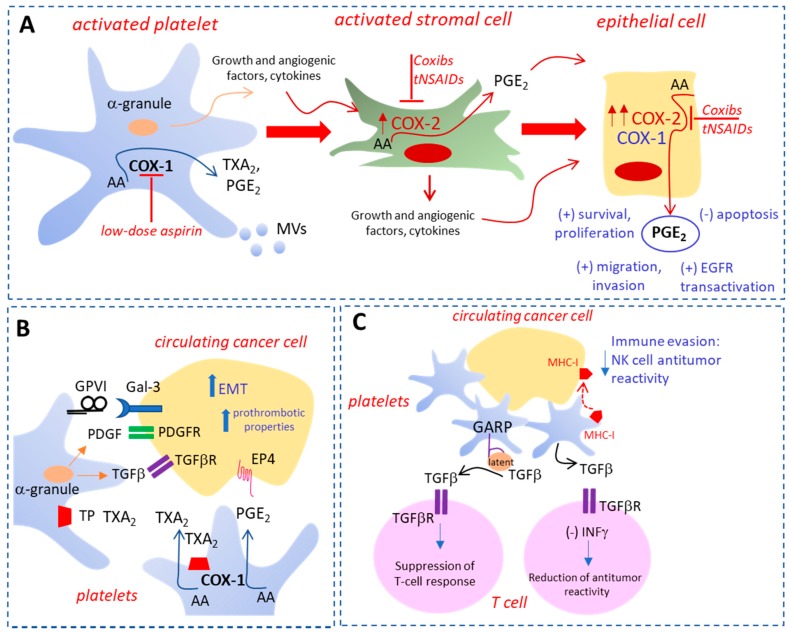
The intimate crosstalk between platelets and cancer. (**A**) In the early phases of colorectal carcinogenesis, activated platelets release soluble factors (including lipids, i.e., PGE_2_ and TXA_2_, and proteins, i.e., SDF-1α, growth and angiogenic factors, cytokines, including interleukin-1β) and MVs, which may, in turn, activate stromal cells, thus further promoting the release of inflammatory mediators and growth factors. Growth, angiogenic factors and cytokines may participate in the induction of COX-2 in stromal cells and epithelial cells. Enhanced levels of COX-2-dependent PGE_2_ in stromal and then epithelial cells contribute to the development of typical hallmarks of cancer, including cancer cell survival, resistance to apoptosis, proliferation, migration, and invasion. Moreover, COX-2-dependent PGE_2_ causes the transactivation of EGFR involved in colorectal cancer development. (**B**) EMT induction in cancer cells is a key mechanism involved in platelet-mediated metastasis formation, and is characterized by reduced levels of typical epithelial markers and increased expression of many mesenchymal markers; also, mesenchymal-like cancer cells have enhanced prothrombotic properties. This leads to the activation of platelets by cancer cells and the release of TXA_2_, which binds to the platelet receptor TP, allowing the amplification of the platelet response. PGE_2_, PDGF, and TGF-β are platelet-derived mediators that mediate the induction of EMT, thus leading to tumor invasion and metastasis formation. (**C**) Platelets promote metastasis by providing cancer cells with protection from immune surveillance due to the so-called “platelet mimicry” phenomenon, characterized by the transferring of platelet proteins to cancer cells, including the MHC-I. The resulting “phenotype of false pretenses” disrupts recognition of tumor cell missing self, thereby impairing cytotoxicity and IFN-γ production by NK cells. Also, GARP activates latent TGF-β, promoting the suppression of immune response to cancer cells mediated by regulatory T cells. Platelet release of TGF-β impairs interferon-γ production and NK cell cytotoxicity. Abbreviations: prostaglandin E_2_, PGE_2_; thromboxane A_2_, TXA_2_; stromal cell-derived factor-1α, SDF-1α; microvesicles, MVs; cyclooxygenase-2, COX-2; epidermal growth factor receptor, EGFR; epithelial mesenchymal transition, EMT; Platelet-derived growth factor, PDGF; Transforming growth factor beta; TGF-β; histocompatibility complex class I, MHC-I; interferon-γ, IFN-γ; natural killer, NK; glycoprotein A repetitions predominant, GARP; arachidonic acid, AA; Nonsteroidal anti-inflammatory drugs, NSAIDs; glycoprotein VI, GPVI; Galectin-3, Gal-3.

Platelets are the cells that respond first to tissue damage to restore the normal functions, but, if uncontrolled, platelet activation supports the development of a chronic inflammatory response that may translate into cancer development [16]. In this scenario, platelets participate in the activation of stromal cells that release inflammatory mediators and growth factors (Figure 1A) [16]. Among them, PGE_2_ production is significantly increased due to the induction of cyclooxygenase (COX)-2 (Figure 1A) [16,17]. Altogether these events translate to the expression of COX-2 in the epithelial compartment associated with enhanced production of prostanoids, including PGE_2_ (Figure 1A) [16,17]. The increased biosynthesis of PGE_2_ promotes cellular proliferation, migration, invasion, angiogenesis, and immunosuppression (Figure 1A) [18,19]. Intestinal epithelial cells overexpressing COX-2 are characterized by higher levels of the anti-apoptotic protein Bcl-2 and by increased resistance to apoptosis [20]. Finally, COX-2-dependent PGE_2_ induces the transactivation of epidermal growth factor receptor (EGFR) (Figure 1A), a transmembrane receptor tyrosine kinase of the Erythroblastic Leukemia Viral Oncogene Homolog ErbB family, involved in the development of colorectal (CRC) [21]. The critical role of COX-2 overexpression in tumorigenesis of CRC [19,22] is sustained by the results of randomized clinical trials (RCTs), showing that selective COX-2 inhibitors (i.e., celecoxib and rofecoxib, which belong to the family of nonsteroidal anti-inflammatory drugs (NSAIDs) reduce the risk of sporadic colorectal adenoma recurrence [23,24,25]. However, the increased risk of CV events associated with the use of these drugs precludes their use for long-term chemotherapy [26]. The same efficacy was found in RCTs using low-dose aspirin, another NSAID that mainly targets platelets [27]. Since the drug, when given at low doses, primarily affects the platelets, it was proposed that platelet activation at the site of tissue damage is the event initiating the cascade of reactions/signaling that promote cell transformation and tumor growth [27]. Interestingly, it was shown that platelet-derived products, such as PGE_2_, the platelet-derived growth factor (PDGF), and the cytokine tumor growth factor (TGF)-β, contribute to the epithelial–mesenchymal transition (EMT) (Figure 1B) [28,29,30]. EMT is a biological process whereby epithelial cells undergo multiple biochemical changes, leading to the acquisition of a mesenchymal cell phenotype characterized by enhanced migratory capacity, invasiveness, and increased resistance to apoptosis [31,32]. This phenomenon is added to others mediated by platelets that promote the development of metastases [33].

Several lines of evidence suggest the role of platelets in metastasis formation via different biological mechanisms [31].

Tumor cells can induce platelet aggregation, a phenomenon called tumor cell-induced platelet aggregation (TCIPA), which represents a recognized event in cancer hematogenous dissemination and an early step in the metastatic process [34,35,36]. The determinants of the interaction between platelets and cancer cells vary depending on tumor type, platelet agonists generated by the tumor cells and microenvironment. A detailed description of the phenomenon of TCIPA is reported in an excellent recent review [36]. It has been proposed that targeting direct molecule contact between platelets and tumor cells may have great potential for new adjuvant antitumor therapies (see Section 3.2 Table 1).

The interaction of platelets with tumor cells reduces the levels of the epithelial marker E-cadherin, and increases the expression of different mesenchymal markers, such as vimentin, fibronectin, Twist, and Snail, which are critical events in the EMT process (Figure 1B) [28,29,30]. These changes lead to enhanced migratory properties and invasiveness of cancer cells (Figure 1B) [28,29,30]. Importantly, mesenchymal-like cancer cells are characterized by enhanced prothrombotic properties, leading to platelet activation and increased production of TXA_2_ and PGE_2_ both in vitro and in vivo (Figure 1B) [29]. The formation of platelet aggregates surrounding the cancer cells in the bloodstream enhances survival and promotes the adhesion to endothelial cells, thus facilitating cancer cell extravasation and the metastatic colonization of distant organs [33]. Moreover, platelets play an immunosuppressive role during tumorigenesis by protecting cancer cells from natural killer (NK) cell-mediated lysis in the circulation and tumor microenvironment [37]. A central role is played by the platelet release of TGF-β, which impairs interferon-γ production and NK cell cytotoxicity [38]. Also, platelets express the glycoprotein A repetitions predominant (GARP), which activates latent TGF-β (Figure 1C) [39]. Platelets induce the “platelet mimicry” of cancer cells, which involves the acquisition of several platelet receptor markers by tumor cells [40] and allows tumors to evade attack from NK cells [40,41]. Interestingly, platelets can transfer their major histocompatibility complex (MHC) class I molecules to tumor cells (Figure 1C) [41]. Furthermore, the formation of cancer cell–platelet–neutrophil complexes may promote cancer immune escape [42,43].

Platelets release MPs into the plasma, and the number is increased in individuals bearing solid tumors [44,45]. Purified platelet-derived MPs can transfer some miRs to cells following co-incubation in vitro, and regulate gene expression [13]. Moreover, profiling of miRs in platelet-derived MPs has been identified as a diagnostic and prognostic tool of malignancy and implicated in therapy resistance [46,47]. However, the effect of platelet-derived MPs in tumorigenesis via the transfer of miRs is still controversial. In fact, Tang et al. showed that in SKOV3 cells (a human ovarian cancer cell line) the selective uptake of MPs in vitro induced EMT via the transfer of miR-939 [48]. Interestingly, they found that secretory phospholipase A_2_ type IIA (sPLA2-IIa) mediated the intake of MPs by SKOV3 cells [48]. Recently, Michael et al. found that platelet-derived MPs infiltrate solid tumors in humans and mice and transfer platelet-derived RNAs, including miRs (miR-24 was a major species), to tumor cells in vivo and in vitro, resulting in tumor cell apoptosis [49]. Thus, further exploration is necessary before the MPs, and miRs delivery will be used as a possible therapeutic approach to fight cancer and metastasis.

## 3. Effects of Antiplatelet Agents in Cancer

### 3.1. Low-Dose Aspirin

Overview analyses of data from over 40 observational studies and the long-term follow-up of 51 RCTs of aspirin, designed to study the prevention of vascular events, have provided the evidence that the drug may reduce the incidence and risk of mortality for several common cancer types, such as CRC, other gastrointestinal cancers and breast cancer [50]. Cancer prevention (death and incidence) by aspirin seems to be unrelated to the dose, and a maximal effect is obtained at low doses (75–300 mg/day) [51]. This effect is similar to that found in the meta-analysis of RCTs of antiplatelet therapy for prevention of death, myocardial infarction, and stroke, in high-risk patients [52]. This evidence sustains the hypothesis that the chemopreventive effect of aspirin is mainly related to its antiplatelet action [27]. The efficacy of low-dose aspirin to prevent death due to CRC is detected at long-term follow-up [7,8,11], thus leading to the proposal that aspirin influences early events of intestinal tumorigenesis through its capacity to affect platelet function and the release of a broad array of mediators and EVs [27]. Additional post hoc analyses of RCTs with aspirin showed that overall cancer incidence was reduced from three years, suggesting a potential effect in reducing the progression of pre-existing cancer and metastasis [10]. Numerous mechanistic studies have been performed in vitro and in vivo using animal models, and the results show that low-dose aspirin prevents metastasis development by interrupting the crosstalk between platelets and cancer cells [27,29].

However, the mechanism of action of low-dose aspirin in the prevention of cancer is still under debate, and a scientific consensus has not been reached yet. The information on the pharmacokinetics (PK) and pharmacodynamics (PD) of aspirin at low doses supports the critical role of the inhibition of platelet function on the anti-cancer effects of the drug. In fact, aspirin causes a persistent inhibition of the biosynthesis of prostanoids through the irreversible inactivation of COX-1 and COX-2 [26,53]. These effects depend on the capacity of the drug to acetylate the cyclooxygenase active site, at Serine-529 and Serine-516 of COX-1 and COX-2, respectively [26,53], even at the low concentrations detected in the systemic circulation after dosing with 100 mg of aspirin (i.e., approximately 4 μM) [54]. However, aspirin has a half-life of only 20 min and, when given once daily, translates into a preferential inhibitory effect towards COX-1 in the non-nucleated platelets, characterized by limited de novo protein synthesis [54]. The irreversible inactivation of platelet COX-1 by low-dose aspirin, which persists throughout the dosing interval (24 h), leads to a virtually complete inhibition (≥97%) of TXA_2_ [55]. The other NSAIDs, which reversibly inhibit COX-isozymes, do not have these features: profound and persistent inhibition of platelet COX-1 between doses [26]. This explains why only low-dose aspirin, among the NSAIDs, confers CV protection [26].

Although the antiplatelet action of low-dose aspirin may explain its chemopreventive effect, recent findings have shown that the drug acetylates COX-1 expressed in colorectal mucosa, even if at a lower extent than in the platelet, leading to incomplete inhibition of PGE_2_ biosynthesis [56] and the inhibition of pro-tumorigenic pathways, such as the phosphorylation of S6 (p-S6) [56]. The formation of p-S6 enhances the affinity of the 40S ribosomal subunit to a subclass of mRNAs, and thus promotes their efficient translation, and regulates cell growth capacity [57].

Preclinical studies have shown that low-dose aspirin and other antiplatelet agents (including ticagrelor, an antagonist of the P2Y_12_ receptor, or DG-041, an antagonist of the PGE_2_ receptor EP3) can prevent in vitro platelet-induced EMT and migration of human colon adenocarcinoma cell line HT29 co-cultured with platelets (Table 1) [29]. Furthermore, the injection of mesenchymal-like HT29 cells, as a consequence of their exposure to platelets in vitro, into the bloodstream of immunodeficient mice, led to an increased number of lung metastasis nodules as compared to the effect caused by the injection of untreated HT29 cells. This effect was associated with enhanced systemic biosynthesis of TXA_2_, mainly derived from platelets [29]. The administration of low-dose aspirin was able to prevent the higher incidence of lung metastasis by HT29 cells exposed to platelets in vitro versus that caused by the injection of untreated HT29 cells. These effects were accompanied by the reduction of platelet activation [29]. Altogether these findings show that aspirin prevents metastasis by controlling “stem cell mimicry” of cancer cells and blunting their pro-aggregatory effect on platelets [29].

### 3.2. P2Y_12_ Receptor Antagonists

The role of naturally occurring adenine-based purinergic compounds in platelet function is highly organized in a coordinated system of membrane receptors and enzymatic chain [74].

Platelets bear two ATP metabotropic, G-protein coupled receptors, namely P2Y_1_ and P2Y_12_ and one ATP ionotropic receptor, P2X_1_ [74]. Co-stimulation of Gq-coupled P2Y_1_ receptors (P2Y-R) and G_i_-coupled P2Y_12_ receptor (P2Y_12_-R) pathways is required for a full platelet aggregation induced by ADP [75], and a sustained P2Y_12_-R activation contributes to thrombus formation. P2X_1_ receptor amplifies the aggregation response to a submaximal concentration of the agonist by a mechanism mainly involving Ca^2+^ influx [76].

The expression of P2Y_12_-R once considered restricted to platelets [77], is also shown in other types of cells including microglia, vascular smooth muscle cells and eosinophils [78,79,80]. Thus, a role in inflammation and immune modulation is suggested for this purinergic receptor [81,82].

On the other hand, the expression of P2Y_12_-R in cancer cells is still poorly studied [83]. P2Y_12_-R has been detected in both glioma and astrocytoma cells, where it drives a proliferative response [84,85]. This purinergic receptor has also been reported in human breast cancer cell lines, including MCF-7 and MDA-MB-231, where its expression is increased under stress conditions, such as serum starvation or cisplatin treatment, thus suggesting a role in chemotherapy resistance [86]. This hypothesis is supported by the evidence that in mouse mammary carcinoma cells 4T1, 2-MeSAMP, a competitive P2Y_12_-R inhibitor, is also able to reduce the cisplatin-mediated increase of hypoxia-inducible factor 1 alpha, a transcription factor involved in the resistance to cytotoxic therapy, and to increase mechanisms of DNA-damage repair [86].

Currently, only P2Y_12_-R antagonists are in clinical use as antiplatelet drugs. These agents, which are the most widely prescribed compounds in CV disease after aspirin, include thienopyridines (ticlopidine, clopidogrel, prasugrel) inhibiting P2Y_12_-R through an irreversible mechanism, and another class of agents, such as ticagrelor, cangrelor, and elinogrel, which act as reversible antagonists [87,88].

In a study performed in an animal model of chronic immune-mediated hepatitis B, which evolved towards hepatocellular carcinoma, the treatment with low-dose aspirin, clopidogrel, or more significantly the combined therapy with the two drugs reduced not only the development of the tumor but also the numbers of deaths [58] (Table 1).

Moreover, in a model of orthotopic ovarian cancer, induced by the injection of the A2780 human ovarian cancer cells into nude mice, the reversible P2Y_12_-R inhibitor ticagrelor diminished the growth of primary tumors when administered by daily gavage [89]. The role of P2Y_12_-R in this kind of cancer is strengthened by the evidence that, when ID8-VEGF murine ovarian cancer cells were injected into the peritoneum of mice with deletion of the P2Y_12_-R^−/−^ mice, tumor growth was reduced by 93% with respect to wild-type animals [89].

P2Y_12_-R seems to play a role also in tumor dissemination. Ticlopidine administered p.o. in a rat model of spontaneous pulmonary metastasis of Lewis lung carcinoma was reported to suppress the dissemination process [59]. Moreover, in animal models of spontaneous or experimentally induced lung metastasis, obtained by injecting Lewis lung carcinoma cells or B16 melanoma cells, respectively, the P2Y_12_-R deficiency was reported to be linked to a reduced weight of metastasis [90].

Recently, in vitro studies have shown that the blockage of the platelet P2Y_12_ receptor by ticagrelor affects EMT and migration induced by the exposure of HT29 cells to platelets [29] (Table 1). These effects were associated with the simultaneous inhibition of platelet TXB_2_ and PGE_2_. Reduced PGE_2_ production in platelet–cancer cell crosstalk may prevent the activation of the PGE_2_ receptor EP4 on HT29 cells, which promotes EMT and migration through the induction of Twist1. In fact, Twist1 is involved in the downregulation of E-cadherin and the upregulation of RAC1 (Ras-related C3 botulinum toxin substrate 1), a small G-protein of the Rho family [29].

Also, ticagrelor has been shown to impair in vitro the interaction of several human mammary cancer cell lines, from the poorly metastatic MCF-7 to more aggressive MDA-MB-468, and MDA-MB-231, with platelets [60] (Table 1). This effect was partially dependent on the ability of ticagrelor to reduce the surface expression of platelet P-selectin induced by ADP, thus affecting the interaction of platelets with P-selectin glycoprotein ligand-1 expressed by the cancer cells [60].

Ticagrelor reduced the lung colony-forming units and improved survival in an orthotopic 4T1 breast cancer model obtained by inoculating 4T1 mammary carcinoma cells into the mammary pad of female BALB/c mice (Table 1). Interestingly, the drug was also able to diminish, in a significant manner, the number of tumor cell-platelet aggregates present in the lung at 10, 30, and 60 min following the intravenous administration of 4T1 cells [60].

Although these findings suggest a role for P2Y_12_-R in mediating platelet–cancer cell crosstalk and provide evidence for the use of P2Y_12_-R antagonists as an additional strategy in chemotherapy, no results from RCTs aimed at assessing their effect on cancer and metastasis are available. On the contrary, some concerns about the safety of the antiplatelet therapy targeting P2Y_12_-R arose from the results of two large RCTs, the Therapeutic Outcomes by Optimizing Platelet Inhibition with Prasugrel—Thrombolysis in Myocardial Infarction (TRITON-TIMI) and the Dual Antiplatelet Therapy (DAPT) trial [91,92,93]. In the first one, an increase in solid tumors was associated with the use of prasugrel, whereas in the second trial, higher rates of death, due to both cancer and trauma, were detected in patients treated with clopidogrel. However, a systematic review and meta-analysis on cancer event rate and mortality following thienopyridine use showed that there is no increased risk with the use of P2Y_12_-R antagonists [94]. These results supported a previous meta-analysis conducted by the Food and Drug Administration of all the long-term RCTs on dual antiplatelet therapy with aspirin and clopidogrel given for 12 months or longer, which stated that the P2Y_12_-R antagonist did not modify the risk of cancer-related deaths [95].

Further mechanistic studies are necessary to clarify the possible contribution of P2Y_12_-R blockage in extraplatelet cells to the anticancer effect of P2Y_12_-R antagonists. Moreover, it is required to verify the potential improvement in efficacy by their co-administration with low-dose aspirin.

### 3.3. Thrombin Receptor Antagonists

Thrombin is a serine protease that exerts a key role in the coagulation cascade by cleaving fibrinogen to yield fibrin and activating platelets, thus ensuring the formation of an effective blood plug. Thrombin’s effects on platelets, in humans, are mediated by two members of PAR family, PAR-1, the primary thrombin receptor, and PAR-4 [96].

Once activated by a peculiar proteolytic mechanism, these receptors couple to different G-proteins, with PAR-1 linking to G_q_, G_i_ and G_12/13_ and PAR-4 activating G_q_ and G_12/13_ [97]. The activation of PAR-1 coupled G-proteins results in the stimulation of different signaling pathways including mitogen-activated protein kinase, Rho kinase, phospholipase C-β and phosphatidylinositol 3-kinase; these factors are known to participate in the modulation of cell proliferation, migration, and adhesion [98].

Interestingly, PAR-1 has also been detected in several types of tumors including melanoma, lung, breast, ovarian, prostate, and gastric cancer [99,100,101,102], and, importantly, its expression levels have been reported to associate with poor prognosis in most of these tumors [99,100,101,102,103].

PAR-1 has been reported to contribute to cancer cell invasion and dissemination through several mechanisms including EMT. In A549 human lung carcinoma cells in vitro, thrombin-activated PAR-1 decreases E-cadherin and increases α-SMA protein expression, and both are markers of the EMT process [104]. Consistently, in MCF-7 cells, a human breast cell line, doxycycline, a tetracycline with recognized anti-tumor activity, constrains EMT by inhibiting the PAR-1/NF-κB/miR-17/E-cadherin pathway [105]. Moreover, in 45% of 129 samples from patients with gastric cancer, intense positive immunostaining for PAR-1 was reported [103]. This expression was associated with depth wall invasion, peritoneal dissemination, and a higher risk of death [103].

In the human gastric adenocarcinoma cells MKN-28 and SNU-638, galectin-3, a carbohydrate-recognition protein involved in cancer cell dissemination, increased PAR-1 expression and cell migration, using a zebrafish embryo in vivo model of cancer cell invasion [106]. These effects were markedly reduced by PAR-1 silencing [106]. Accordingly, a parallel increase in galectin-3 and PAR-1 expression was detected in malignant tissues from gastric cancer patients when compared to normal samples. Also, the two proteins were found to co-localize only in the cancerous areas of the tissue [106].

Beyond platelets and malignant cells, the expression of PAR-1 is detected in fibroblasts, macrophages, and endothelial cells, which represent the principal cell types of the tumor microenvironment deeply involved in cancer cell seeding and growth [107].

It has been reported that the subcutaneous injection of MC38 cells, an aggressive C57Bl/6-derived colonic adenocarcinoma cell line, in PAR-1^−/−^ and wild-type mice caused the development of palpable tumors in both genotypes [108]. However, the tumors grew significantly more slowly in PAR-1^−/−^ mice than in control animals [108]. These data suggest a role for stromal-PAR-1 in tumor outgrowth. However, experiments with conditional animal models in which PAR-1 gene expression is “floxed,” and thus silenced in a cell-specific manner, are needed to corroborate this mechanism.

Although in vitro and in vivo results suggest that the control of PAR1-mediated signaling may represent a promising strategy for the treatment of malignancy, currently only vorapaxar (SCH530348) is approved, as a PAR-1 antagonist, for patients with a history of myocardial infarction or peripheral arterial disease in the United States and with a history of myocardial infarction in Europe [109]. The clinical development of atopaxar (E5555), another PAR-1 antagonist, is limited to phase 1 and phase 2 trials [110].

It has been recently reported that vorapaxar pretreatment of three different epithelial ovarian cancer cells (SKOV-3, OVCAR-3, and CaOV-3) can reduce the thrombin-induced cell proliferation to the basal values (Table 1). It does not seem to be an off-target effect given that, in the absence of thrombin, vorapaxar was unable to modify the baseline levels of cell proliferation [61].

Despite the strong rationale and these encouraging effects on cancer cells in vitro, the potential use of these drugs in long-term treatments in chemoprevention or chemotherapy seems to be not viable due to their possible side effects, including the significantly increased risk of bleeding [109].

Finally, PAR-1 may be of interest for the development of a novel approach to pathological conditions not directly linked to tumorigenesis or cancer cell dissemination, but associated with cancer therapy such as the intestinal radiation injury. Interestingly, in a rat model of intestinal radiation-induced mucositis, the short-term administration of SCH602539, a vorapaxar analog, was effective at reducing early inflammatory and proliferative effects [111].

### 3.4. Glycoprotein IIb/IIIa Antagonists

Integrins represent a broad family of transmembrane adhesion receptors involved in cell–cell and cell–extracellular matrix interactions. Among them, glycoprotein (GP) IIb/IIIa (also known as αIIbβ3) is the most abundant receptor expressed in platelets. On the GPIIb/IIIa receptor, two main binding sites are present. One recognizes the amino acid motif Arginyl-glycyl-aspartic acid (RGD) found on multiple ligands, including fibronectin, von Willebrand factor (vWf), vitronectin and fibrinogen. The other binding site interacts with fibrinogen via the peptide sequence lysine-glutamine-alanine-glycine-aspartic acid-valine (KQAGDV) [112]. GPIIb/IIIa participates in hemostasis and thrombosis by the crosslinking of neighboring platelets mainly through the binding of fibrinogen [113,114]. In fact, fibrinogen contains two RGD sequences and the KQAGDV sequence [115]. However, fibrinogen-independent platelet aggregation in vitro and in vivo has been described [116,117,118] and may play a role in TCIPA [34].

In vivo studies have demonstrated that the inhibition of platelet GPIIb/IIIa could restrain lung colonization of cancer cells [119,120]. Interestingly, a role for platelet GPIIb/IIIa in bone metastasis was also pointed out. In fact, in a mouse model of osteolytic bone metastasis, obtained through the injection of B16 melanoma cells into the left cardiac ventricle, the bone lesions were developed by only 4% of β3integrin^−/−^ mice compared to 74% of β3integrin^+/+^ mice [121]. Moreover, the pharmacological inhibition of murine GPIIb/IIIa by ML464, orally administered to the β3integrin^+/+^ before the B16 cell injection, markedly reduced the number of bone metastasis and also the number and size of visceral metastasis [121].

More recently, it has been reported that the use of JON/A, a blocking antibody against GPIIb/IIIa, can markedly reduce the adhesion of B16 melanoma cells in vitro to immobilized murine platelets without affecting their number [122]. A role of this integrin in platelet–cancer cell interaction has been shown in melanoma cells (M3Dau cell line) where a GPIIb/IIIa-like integrin was reported to guarantee a direct binding with the platelet GPIIb/IIIa counterpart [123]. Thus, even though the role of GPIIb/IIIa in the platelet–cancer cell crosstalk has to be further clarified, this integrin represents an attractive chemotherapeutic target.

Currently, three GPIIb/IIIa blockers, namely abciximab, eptifibatide, and tirofiban, are used in clinical practice to prevent ischemic events in high-risk patients [124,125,126].

Up to now, few in vitro studies have shown the capability of these agents to affect cancer cell proliferation and migration. Tirofiban and eptifibatide have been reported to constrain tumor cell invasive potential in HSC-3 human tongue squamous cell line and human breast carcinoma MDA-MB231 cells, respectively (Table 1) [63,126]. Abciximab and eptifibatide have been shown to cause apoptosis in MCF-7 human breast cancer cells [62] (Table 1).

All these drugs can be administered only by intravenous injection and enhanced risk for serious adverse events, such as bleeding, is associated with their use [127]. These factors may limit their long-term use as chemotherapeutic agents.

Unfortunately, the attempt to develop GPIIb/IIIa blockers administrable p.o., such as lotrafiban, xemilofiban, orbofiban, and sibrafiban, have shown, in all the undertaken clinical studies, an increased risk of mortality associated with their use [128].

As a consequence of these negative outcomes, the development of further oral GPIIb-IIIa antagonists was abandoned, but the interest towards a therapeutic strategy involving this integrin was not. Thus, novel blockers were designed to bind only the activated GPIIb/IIIa receptors.

A human single-chain antibody scFv MA2 has been developed that is unable to cause conformational changes but can inhibit fibrinogen binding to platelets. The novel compound has been tested in an animal model of thrombosis by using C57BL/mice in which filter paper saturated with ferric chloride was positioned under the right carotid artery for 3 min [129]. It showed an antithrombotic potency similar to tirofiban and eptifibatide when infused through the tail vein before the ferric chloride treatment, without significantly prolonging bleeding time [129].

Although no data are yet available on the potential effects of scFvMA2 in tumorigenesis, this compound can be used as a novel diagnostic tool. Indeed, an scFv that can bind to the active conformation of GPIIb/IIIa in mouse, or human platelets, has been conjugated with different types of contrast agents for fluorescence, PET, and ultrasound imaging (namely Cy7, ^64^Cu and ultrasound-enhancing microbubbles). Irrespective of the coupling tracer, this innovative approach allowed us to individuate activated platelets within the tumor microenvironment with high specificity, and sensitivity and give an accurate anatomical view of the tumor itself. The study was carried out in four different human tumor xenograft mouse models, including SKBr3 and MDA-MB-231 breast cancer, HT-1080 fibrosarcoma, and Ramos Burkitt’s lymphoma [130]. Further studies are needed using these GPIIb/IIIa-based contrast agents to confirm their possible use as an auxiliary non-invasive method for the detection and imaging of cancer and metastatic lesions.

## 4. Novel Antiplatelet Agents in Clinical Development

### 4.1. GPVI Blockers

GPVI is the principal human platelet collagen receptor and is involved in platelet recruitment in response to vascular injury [131]. The interaction of platelet GPVI with immobilized collagen in the extracellular matrix initiates platelet signaling pathways essential for platelet activation and thrombus formation [131]. Moreover, GPVI ligation mediates a panel of platelet responses including platelet spreading, granule secretion and integrin α_IIb_β_3_-dependent aggregation [131].

The use of anti-GPVI antibodies or the soluble GPVI receptor has been proposed to inhibit the interaction of collagen with platelet GPVI [132]. Revacept, a novel antiplatelet agent in clinical development, is a fusion protein of the dimeric form of the soluble GPVI receptor with the Fc immunoglobulin component, which binds to collagen at the sites of vascular injury. Thus, revacept may prevent platelet adhesion and consecutive thrombus formation at the site of vascular injury [132]. In a clinical phase I study, revacept inhibited collagen-induced platelet aggregation in a dose-dependent fashion while not affecting ADP- or thrombin-dependent platelet aggregation [133]. Interestingly, the agent did not affect general hemostasis, as determined by measuring bleeding times, or coagulation (assessed by evaluating activated partial thromboplastin time and international normalized ratio) [133]. Results from phase II clinical studies are ongoing to determine the efficacy and the safety profiles of revacept in patients with symptomatic carotid artery stenosis, transient ischemic attacks or stroke, and in coronary artery disease patients [131].

In preclinical studies performed in vitro, revacept interfered with the interaction of platelets and colorectal cancer cells HT29, thus preventing the induction of COX-2 (considered a pivotal event in carcinogenesis) and EMT [30]. COX-2-dependent PGE_2_ biosynthesis caused the downregulation of p21^WAF1/CIP1^ and the upregulation of cyclinB1, since these effects were prevented by rofecoxib (a selective COX-2 inhibitor) and rescued by exogenous PGE_2_ [30]. Galectin-3, which contains a collagen-like domain, was involved in the platelet-dependent induction of COX-2 in HT29 cells [30]. In fact, inhibitors of galectin-3 function [β-lactose, a dominant-negative form of galectin-3 (Gal-3C), and anti-galectin-3 antibody M3/38] prevented the aberrant COX-2 expression. A similar result was obtained by revacept. These findings support the role of galectin-3 and collagen receptors in platelet–cancer cell crosstalk [30]. These results reveal that blockers of collagen binding sites, such as revacept, and galectin-3, may represent an innovative strategy in colon cancer chemotherapy that should be tested in experimental animals.

Consistent with the role of platelet GPVI in metastasis formation, it has been shown that in GPVI deficient mice, the injection of Lewis Lung carcinoma (D21) or melanoma B16F10.1 cells caused a reduction of about 50% in the lung number of tumor foci compared to control wild-type mice [134].

### 4.2. Antagonists of the EP3 Receptor for PGE_2_

PGE_2_ activates the platelet receptors EP2, EP3, and EP4, which have opposite effects on adenylate cyclase: EP3 inhibits, whereas EP2 and EP4 activate the enzyme [49]. Some lines of evidence show that EP3-induced inhibition of adenylate cyclase predominates over EP2 and EP4 activations [135,136]. PGE_2_ alone does not cause platelet aggregation, but sensitizes the platelets to aggregate in response to different activators [136]. Deletion of EP3 on platelets reduced in vivo murine atherothrombosis [137]. Furthermore, the blocking of EP3 decreased murine pulmonary embolism and potentiated platelet inhibition by clopidogrel without altering tail bleeding time [138]. In healthy individuals, the selective EP3 antagonist DG-041, in clinical development (phase II), reduced platelet aggregation without significantly altering the cutaneous bleeding time [138]. Thus, targeting the EP3 receptor might enhance the antiplatelet effects of conventional antithrombotic agents without increasing the bleeding risk.

In platelet-cancer cell co-cultures, DG-041 can prevent platelet-induced EMT and enhanced migratory capacity of HT29 colon cancer cells [27]. Since, EP3 receptors were expressed in platelets, but not in HT29 cells, these effects of DG-041 were mediated by the selective blockage of platelet EP3 [29]. The efficacy of EP3 antagonism in the prevention of tumorigenesis and metastasis has to be proved in appropriate animal models before testing them in patients.

### 4.3. GPIbα Antagonists

GPIbα is a glycoprotein component of the platelet GPIb-IX-V complex that mediates adhesion to vWf normally present in the vascular subendothelium. This event induces platelet adhesion and platelet aggregation, particularly at high shear. Some blockers of the GPIbα are in preclinical or clinical development as antithrombotic agents (such as the snake venom-derived antagonist anfibatide and humanized anti-glycoprotein Ib monoclonal antibody (h6B4-Fab)) [139] (Table 1).

Some reports have shown that blocking GPIbα may inhibit TCIPA and tumor arrest in the vasculature [36]. However, other studies have found no impact of antibodies against GPIbα on TCIPA [140]. Moreover, some discrepancies have been reported in an experimental metastasis model using B16F10 melanoma cells in mice. In fact, GPIbα deletion was associated with a lower number of lung metastases than wild-type mice, whereas functional inhibition of GPIbα by monoclonal antibodies caused a strong increase in pulmonary metastasis [68]. However, in the presence of P-selectin deficiency, GPIbα blockade had no enhancing effect on metastasis. These results suggest the involvement of GPIbα in the induction of metastasis by P-selectin [68].

### 4.4. P-Selectin Inhibitors

P-selectin (CD62P) is a protein stored in granules of platelets and endothelial cells, i.e., α-granules and Weibel–Palade bodies, respectively, that mediates the interaction of activated endothelial cells or platelets with leukocytes [141]. Platelet activation is associated with P-selectin translocation to the cell surface and the formation of platelet–monocyte aggregates, which promote vascular inflammation, thrombosis, but also metastasis [142]. Platelet interactions with cancer cells, including colorectal adenocarcinoma cell line Caco2 [143] and ovarian tumor epithelial cell line 59 M [144], have been shown to be associated with the enhanced expression of P-selectin on platelets. P-selectin deletion significantly suppresses the growth of subcutaneously implanted human colon carcinoma cells and lung metastases from intravenously injected cells [145]. Altogether these results sustain the contribution of P-selectin in metastasis and provide the rationale to develop anticancer strategies by targeting the P-selectin pathway.

Anti P-selectin antibody and anti-CD24 (a sialoglycoprotein that binds P-selectin) antibody FL80 are in the preclinical stage of development [69].

We have tested the role of P-selectin in the overexpression of COX-2 in colorectal cancer HT29 cells by the crosstalk with human platelets. However, we obtained negative results using the P-selectin antagonist (gallolyl-*N*-gaba-WVDV-OH) [30]. These results suggest that the direct interaction of platelets with cancer cells is a complex phenomenon and different cancer cell types have developed specific pathways.

## 5. Concluding Remarks

The results of a large number of preclinical and clinical studies with antiplatelet agents and the availability of genetically modified mouse strains with defects in specific platelet proteins have allowed for identifying novel roles of platelets in tumorigenesis and metastasis. Platelets play critical roles in these settings for their capacity to release a wide array of biologically active soluble factors, i.e., lipids and proteins, and vesicles rich in genetic materials, including miRs, which may deliver their cargo to other cells, including cancer cells (Figure 1) [13,27,33,146]. Thus, the platelet is now considered an essential element in the intercellular communication. Novel platelet functions involve the capacity to activate different pathways in cancer cells, resulting in their transition to an invasive mesenchymal-like phenotype characterized by enhanced metastatic potential. Importantly, platelet and platelet-derived MPs may contribute to the immune escape of cancer cells [16]. Moreover, activated platelets play a role in the development of cancer by influencing the early steps of the disease, such as the promotion of chronic inflammation [13,16,17,27,146]. In this context, platelet activation in response to tissue damage leads to the development of a healing program through their adhesion to injured tissues, the release of several factors involved in angiogenesis, and the recruitment of inflammatory and immune cells [16]. The expression of COX-2 in the stromal cellular components amplifies the inflammatory response, which promotes the epithelial cell transformation associated with elevated biosynthesis of COX-2-dependent PGE_2_ [16] (Figure 1). In this scenario, antiplatelet agents inhibit the platelet contribution to tumorigenesis and metastasis development [27].

Although clinical evidence of the anticancer effects by antiplatelet agents is mainly related to the use of low-dose aspirin, a similar efficacy can be assumed for other antiplatelet agents, in particular clopidogrel and other P2Y_12_ antagonists. However, their effectiveness should be tested in population-based case-control studies and RCTs. Importantly, the possible improved anticancer effect by the co-administration of low-dose aspirin with P2Y_12_ antagonists remains to be explored.

Additional evidence for the chemopreventive effects of aspirin is being sought prospectively in ongoing primary prevention trials [27,147]. Moreover, several adjuvant trials of various low-dose aspirin regimens have recently been initiated in patients with newly diagnosed cancers, including colorectal, gastroesophageal, breast, and prostate cancer (e.g., the Add-Aspirin trial) [27].

The results of basic and preclinical research have identified novel platelet targets to fight against cancer development [36]. However, the development of innovative pharmacological approaches for cancer prevention, which involve the chronic use of drugs for a long time, should have two essential features: (i) associated with reduced side-effects and (ii) appropriate bioavailability after oral administration. These criteria are met by aspirin [27]. In fact, the chronic use of aspirin, even at low doses, can be associated with enhanced risk of bleeding [27]. However, the extent of risk reduction of both vascular events and cancer translates into an advantage [27]. Thus, the USA. Preventive Services Task Force recommended initiating low-dose aspirin use for the primary prevention of cardiovascular disease and colorectal cancer (and possibly other cancers) [148].

An emerging field of clinical research is related to the discovery of biomarkers to identify those subjects who will respond to the antineoplastic effect of aspirin. In this context, genomics, transcriptomics, and proteomics information of tumor-associated blood platelets and possibly MPs have the potential to address this essential medical need [13]. In fact, cancer may alter the RNA profile of blood platelets that provide specific information on the location and molecular composition of the primary tumor [149,150,151].

## Figures and Tables

**Table 1:** Effects of antiplatelet agents on tumorigenesis and metastasis formation: in vitro and in vivo studies.

Drug Class	Drug Target	Agents	In Vivo and in Vitro Studies	Reported Effects
NSAIDs	Platelet COX-1	Low-dose aspirin	In vitro co-culture of platelets and human colon adenocarcinoma cell line HT29 [29] HT29-induced hematogenous metastasis in vivo [29] In vivo mouse model of chronic hepatitis B [58]	Prevention of platelet-induced EMT and migration (disruption of cancer cell metastatic potential) [29] Prevention of platelet-induced metastatic and prothrombotic phenotype [29] Prevention of immune-mediated liver injury and fibrosis and HCC development (in combination with clopidogrel) [58]
Thienopyridines	ADP receptor P2Y12	TiclopidineTicagrerol	In vivo model of spontaneous lung metastasis [59] In vitro co-culture of platelets and HT29 colon cancer cells [29] In vitro co-culture of platelets human breast cancer cell lines (MCF-7, MDA-MB-468, and MDA-MB-231) [60] Orthotopic 4T1 breast cancer model [60]	Suppression of metastasis dissemination [59] Prevention of platelet-induced EMT and migration (disruption of cancer cell metastatic potential) [29] Prevention of platelet–cancer cell crosstalk [60] Reduction of metastasis formation and number of tumor cell-platelet aggregates and improvement of survival [60]
PAR-1 antagonists	Protease-activated receptor PAR-1	Vorapaxar	In vitro studies with human ovarian cancer cells (SKOV-3, OVCAR-3 and CaOV-3) [61]	Reduction of PAR-1 agonist-mediated effects including cell proliferation [61]
Glycoprotein IIb/IIIa antagonists	Glycoprotein (GP) IIb/IIIa	AbciximabEpitifabideTirofiban	MCF-7 breast cancer cells [62] In vitro co-culture of thrombin-activated platelets and human breast carcinoma MDA-MB-231 cells [62] MCF-7 breast cancer cells [62] In vitro studies with highly invasive human tongue squamous carcinoma cell line HSC-3 [63]	Tumorigenesis and metastasis control [62] Constriction of tumor cell invasive potential [62] Tumorigenesis and metastasis control [62] Inhibition of promigratory effect induced by Col15 [63]
GPIb inhibitors	Platelet GPIb	Anfibatide anti-GPIbα antibody (h6B4-Fab, GPG-290, and anti-GPIbα)	In vitro and in vivo murine models of thrombosis [64] and phase II human clinical trials [65] High shear arterial thrombosis model in baboons [66] Canine model of artery thrombosis [67] In vivo metastasis model B16F10 melanoma cells [68]	Inhibition of platelet adhesion, aggregation and thrombus formation, without increasing bleeding time [64,65] Reduction of thrombus formation at an injured femoral artery site [66] Prevention of coronary artery thrombosis [67] Promotion of melanoma metastasis [68]
P-selectin (CD62P) inhibitors	Platelet P-selectin and tumor P-selectin ligands	Anti-P-selectin antibody (GA-6), P-selectin Mab, anti-CD24 (P-selectin ligand) antibody FL80	Prostate cancer cell line DU145 [69] Mucin-type ligands bearingsialyl-Lewis X small-celllung cancers, colon cancer and neuroblastoma [70,71] Colon cancer cells MC-38 expressing sulfatedgalactosylceramide-typeligands [72] Murine model of gastric cancer [73]	Prevention of platelet binding to prostate cancer cells [69] Prevention of P-selectin adhesion of platelets to cancer cells [70,71] Prevention of P-selectin-mediated metastasis progression [72] Reduction of gastric cancer metastasis [73]
GPVI antagonists	Platelet GPVI	Revacept	In vitro co-culture of platelets and human colon adenocarcinoma cell line HT29 [30]	Prevention of platelet-induced COX-2 upregulation and EMT [30]
EP3 antagonist	PGE_2_ receptor EP3	DG041	In vitro co-culture of platelets and human colon adenocarcinoma cell line HT29 [29]	Prevention of platelet-induced EMT and migration (disruption of cancer cell metastatic potential) [29]
